# Properties of adaptive, cluster-randomised controlled trials with few clusters: a simulation study

**DOI:** 10.1186/s13012-025-01443-6

**Published:** 2025-07-01

**Authors:** Erin Nolan, Joshua Dizon, Christopher Oldmeadow, Elizabeth Holliday, Alix Hall, Daniel Barker

**Affiliations:** 1 School of Medicine and Public Health, The University of Newcastle, Callaghan, NSW Australia; 2 Hunter Medical Research Institute, New Lambton Heights, New South Wales, Australia; 3 National Centre of Implementation Science, The University of Newcastle, Newcastle, NSW Australia; 4 Hunter New England Population Health, Hunter New England Area Health Service, New South Wales, Australia

**Keywords:** Adaptive designs, Implementation, Bayesian, Optimisation, Simulation

## Abstract

Trials optimising implementation strategies are complex, assess multicomponent strategies, and cluster randomise. We define optimisation as identifying the best combination of components for multi-component implementation strategies. Multi-arm, fixed, cluster randomised control trials (cRCTs) can assess multiple implementation components but suffer from low power due to challenges of recruitment. Adaptive designs offer increased efficiency, when compared to “fixed trial” approaches. A simulation study was conducted to assess whether adaptive designs are feasible (acceptable operating characteristics and adaptive interim decisions) for implementation cRCTs with few clusters. A four-arm cRCT was simulated under varying trial properties. The trials were simulated using fixed design and adaptive design parameters (number of interim analyses, timing of interim analysis, actions at interim e.g. allowing for early stopping for futility, arm dropping) and modelled using Bayesian hierarchical models. The power and type 1 error were compared between the fixed and adaptive designs, and the number of correct interim decisions under the adaptive design were examined. When the intra-class correlation (ICC) was high, the proportion of trials that incorrectly dropped the most effective arm increased. There were small power gains for adaptive designs, without increasing type 1 error. Power gains attenuated when ICC was high and sample size was low. Type 1 error was lower comparable between adaptive and non-adaptive designs. Adaptive designs are feasible for cRCTs with few clusters. They are not as feasible when the ICC is high due to increased risk of incorrect adaptive interim decisions.

**Supplementary Information:**

The online version contains supplementary material available at 10.1186/s13012-025-01443-6.

Contributions to the literature

- Adaptive designs may offer increased efficiency compared to “fixed trial” approaches, Especially for trials aiming to optimise implementation strategies.
- Our study identified that adaptive designs have acceptable operating characteristics and make the correct adaptive interim decisions for trials aiming to optimise implementation strategies that have few clusters.
- Adaptive designs are not as feasible for outcomes that may have a high level of intra-class correlation, due to an increased risk of incorrect adaptive interim decisions.

## Background

Implementation science aims to bring evidence based best practice, frequently a combination of interventions, into standard care. The benefits of interventions and implementation strategies often attenuate or disappear entirely when applied in a real-world setting [1], potentially because real-world constraints such as cost and staff time are not considered at the time of evaluation. This has led to a recent focus on improving interventions and implementation strategies while considering the real-world constraints, a process known as optimisation [2]. Optimisation is defined as “a deliberate, iterative and data-driven process to improve a health intervention and/or its implementation to meet stakeholder-defined public health impacts within resource constraints” [2]. For the focus of this paper the objective of optimisation is to determine which components or combination of components are the most effective within given constraints in a multi-component intervention or implementation strategy.

Trials seeking to optimise implementation strategies are often complex, using clustered randomisation, assessing multicomponent strategies, and with the optimisation process typically conducted over multiple successive trials [3–5]. Cluster randomised trials randomise whole clusters to a treatment arm. This approach to randomisation is often due to the risk of treatment contamination or because the intervention (or its implementation) may only be deliverable at a cluster level (e.g. school policy). In cluster randomised trials, participants within a cluster are not independent from one another. The magnitude of correlation between participant values within a cluster is quantified using the intra class correlation (ICC). The higher the ICC, the lower the effective sample size and thus the power to detect a given effect [6].

More efficient trial designs than consecutive two-arm RCTs are recommended for evaluating whether optimisation was successful, such as multi-arm and factorial designs [7]. These designs allow for researchers to examine the effects of the multiple components of an intervention or implementation strategy [8, 9]. However, guidance in using these designs is aimed towards trials that randomise at the participant level, rather than the cluster level. In addition, many implementation trials have limited power to test the effects of all components and their interactions [10–12]. This limitation, compounded with the lowered power of clustered randomisation, can lead to trials being unable to effectively evaluate the optimisation process, despite best recruitment efforts.

Adaptive designs, unlike fixed trial designs, can update specified design parameters during the trial, based on accumulating data [13]. There are a wide range of adaptations that can be made, with common adaptions including early stopping, response adaptive randomisation, arm dropping, and sample size re-estimation. Adaptive designs have demonstrated benefits for multi-arm clinical trials [13]. A trial re-execution by Ryan et al*.* published in 2020 [14] concluded that using adaptive designs for the four arm Collaborative Ankle Support Trial [15] would have increased power while maintaining appropriate type 1 error and reached a trial conclusion sooner. The benefits of adaptive designs for multi-arm trials, particularly increased power and efficiency, could extend to multi-arm implementation trials, thus increasing their usability as a recommended design when the research goal is optimising an intervention. Factorial designs are also recommended for optimisation [7]. There is existing guidance for the use of adaptive factorial designs, but it is limited mainly to individually randomised trials [16, 17]. Also, implementation scientists have avoided the use of factorial designs stating the design was prohibitively complex [18].

However, not all adaptive designs that have demonstrated benefits for clinical trials may be useful for implementation trials. For adaptive designs to offer benefits for implementation trials, careful consideration must be given to design features common in implementation trials, to ensure the designs and potential adaptations are appropriate. Not every trial can collect a large number of clusters and people per cluster. It’s common for implementation trials to have a low number of clusters [19, 20], with some as low as 12 clusters in total [5]. Trying to allocate those clusters in a multi-arm trial could result in as few as three to four clusters per arm (in a four-arm trial). Whether adaptive designs can be effectively used in such cases has not been explored. Decision-making based on interim analyses in trials with a limited number of clusters or participants may lead to an elevated risk of incorrect interim decisions, such as discarding the most effective arm. For this reason, research is required to assess whether the effectiveness and statistical feasibility of adaptive designs extends to very small implementation optimisation trials.

In addition to the unknown appropriateness of adaptive designs for trials aiming to optimise implementation strategies, there may be potential difficulties modelling the results of these trials. A study by Li that explored whether models could analyse small cluster RCTs found that no modelling methods tested performed well below 10 clusters per arm in the trial [21]. A separate study by Leyrat et al*.* [22] found that a modelling method that performed best to analyse results from small trials, and was recommended previously [23], still failed to converge up to 40% of the time. Modelling the interim analysis for adaptive designs requires finding a method that results in model convergence and performs acceptably when the number of clusters is very low. There has been growing literature for using Bayesian adaptive designs in cRCTs [24–26]. This literature focused on clinical trials, multi-arm or two-arm, with a moderate to large number of clusters per arm. The simulation by Harari et al*.* [24] found that in a large (maximum 550 clusters) three-arm cRCT with an arm dropping design, the proportion of clusters recruited into futile arms decreased dramatically. How these designs extend into trials with a small number of clusters per arm in a multi-arm implementation trial, and how they can be used to find an optimal treatment, is unknown.

Using adaptive designs in trials aiming to optimise the implementation of interventions remains largely unexplored, and decisions in the interim analysis may greatly influence trial outcomes. While adaptive designs can, in general, enhance statistical power and improve efficiency, it is not known how these designs will function with the designs recommended for optimisation, or in the presence of design features common in implementation trials.

The impact on power and type 1 error for various plausible adaptations in an adaptive design for small, multi-arm, cluster randomised implementation trials has not been thoroughly investigated. We propose a simulation study to explore the operating characteristics of various combinations of adaptive trial parameters. Before considering how they would be impacted with different constraint considerations we must first determine if adaptive designs are statistically feasible for trial designs recommended for optimisation. Adaptive designs would demonstrate statistical feasibility if the models converge successfully, and the interim decisions are not frequently incorrect.

## Methods

The ADEMP (aims, data generating mechanisms, estimands, methods, performance measures) [27] and Bayesian simulation study (BASIS) frameworks [28] were used to guide the structure of the simulation study to assess the performance of adaptive designs under varying trial properties.

### Aims and outcomes

The aims of this simulation study were as follows:

1. Assess whether adaptive designs (stopping for futility and arm dropping) are statistically feasible for optimisation trials with small sample sizes and with properties that resemble implementation trials.
2. Identify how adaptive designs affect the power and type 1 error of trials seeking to optimise implementation strategies.

To achieve these aims we performed a simulation study using a Bayesian modelling framework. We chose to do this because while frequentist models may produce potentially biased results or fail to run in trials with small cluster and participant numbers, Bayesian models can yield valuable insights with fewer participants and clusters [29]. The Bayesian inferential paradigm is also very commonly used in adaptive trials [30].

The power and type 1 error of trials both with and without adaptive designs included were examined. These were examined over a range of trial properties reminiscent of trial properties commonly found in implementation trials in designs frequently used for optimisation (multi-arm trials). We must determine whether the models can regularly achieve convergence in this space and how often the correct adaptive interim decision is made from models that do converge. To assess this, the convergence and adaptive interim decisions were examined.

### Data generating mechanisms

The simulation study was designed to evaluate the most preferred adaptive designs identified from advice with experts in the field and trial properties found commonly in implementation trials. The basic design was a four-arm, cluster RCT, with enrolment consisting of an entire cluster at a time. Arm one was a control, and arms two, three, and four were treatment arms.

Simulations were performed for this design under a fixed trial framework, and also using an adaptive design. Simulations varied those trial properties that differ among implementation trials and which impact power, type 1 error, and convergence. These properties were the effect size, intra-cluster correlation (ICC), number of participants per cluster (n), and number of clusters per arm (k). The outcome of the trial was assumed to be binary (taking values of 0/1) following a binomial distribution, with a higher proportion of events being favourable. A fully factorial combination of trial properties was generated. Table 1 details the trial properties that were simulated, along with the justification for each property.

**Table 1:** Trial properties along with justification

Property	Level	Justification
Effect size	Scenario 1	One treatment arm is more clearly the optimal arm compared to the other treatment arms, with a larger difference in the effect size between treatment groups
	Scenario 2	One treatment arm is the optimal arm, but it is not as clear, with a smaller difference in the effect size between the treatment groups
	Scenario 3 – No effect	To examine the type 1 error
Intra-cluster correlation (ICC)	0.05	A smaller ICC that is still plausible for clusters in implementation trials [55]
	0.1	
	0.2	A larger, plausible ICC seen in implementation trials [43]
Number of participants per cluster (n)	5	Plausible when the outcome level is staff (e.g. teachers, doctors) [56]
	25	
	50	Plausible when the outcome level is benefactors of the treatment (e.g. students, patients)
Number of clusters per arm (k)	5	The lower bound of the number of clusters per arm possible in a frequentist framework [57]
	10	Plausible for larger scale trials [56]

Let ${Y}_{imj}=0$ represent an undesirable outcome and ${Y}_{imj}=1$ represent a desirable outcome for the *ith* participant in the *mth* cluster in the *jth* arm.

$${Y}_{imj}\sim Bernoulli\left({p}_{mj}\right)$$

$$\text{log}\left(\frac{{p}_{mj}}{1-{p}_{mj}}\right)= {\beta }_{0}+{X}_{mj}{\beta }_{j}+{\alpha }_{m}$$

$${\alpha }_{m}=\sqrt{\frac{ICC*\frac{{\pi }^{2}}{3}}{1-ICC}}$$

where ${p}_{mj}$ is the probability that a participant had the event of interest, ${\beta }_{0}$ is the intercept, ${X}_{mj}$ is the variable indicating treatment arm, ${\beta }_{j}$ is the effect of the treatment arm, and ${\alpha }_{m}$ is the cluster level random effect.

The simulation considered three scenarios to assess the trial designs over the property space. Scenarios 1, 2, and 3 had a clearer optimal arm, a less clear optimal arm, and no effect, respectively (Table 2). Arm four was always the arm with the highest treatment effect, and thus the optimal arm in the trial.

**Table 2:** Proportion of favourable events by arm and scenario

	Proportion of favourable events (${Y}_{imj}$ = 1)		
Arm	Scenario 1 (Clearer optimal arm)	Scenario 2 (Less clear optimal arm)	Scenario 3 (no effect)
Arm 1 (Control)	0.1	0.1	0.1
Arm 2	0.2	0.3	0.1
Arm 3	0.4	0.4	0.1
Arm 4	0.6	0.5	0.1

### Estimand

Let $\theta$ represent the log odds ratio of the favourable outcome (Y = 1) vs the unfavourable outcome (Y = 0). $\beta$ is related to $\theta$ as follows:

$$\begin{aligned}{\beta }_{0}&= {\theta }_{1}\\{\beta }_{j}&={\theta }_{j+1}-{\theta }_{1};\text{for j } \text{in }1, 2, 3\end{aligned}$$

### Simulation

All models were run using a Bayesian framework. Hierarchical models were run in ‘Stan’ and the output sent to ‘R’ via ‘cmdstanr’ [31–33]. Weakly informative priors used for the treatment arm parameters ${\theta }_{j}$ for $j=1, 2, 3, 4$, and the hyperprior specification for cluster parameters ${\alpha }_{m}$ are defined in (Eq. 1). These priors represent a balance between being informative enough to assist model convergence, but remaining only weakly informative.

1 $$\begin{aligned}{c}{\{{\theta }_{1},\theta }_{2},{\theta }_{3},{\theta }_{4}\} &\sim N\left(0, 2\right)\\ {\alpha }_{m} &\sim N\left(0,{\sigma }_{a}\right)\\ {\sigma }_{a} &\sim halfNormal\left(0, 0.2\right)\end{aligned}$$

The complete Bayesian model specification was:

$$Bernoulli\left(Y|logi{t}^{-1}\left({\theta }_{1}+{{W}_{m}\alpha }_{m}+{{X}_{m2}\theta }_{2}+{X}_{m3}{\theta }_{3}+{X}_{m4}{\theta }_{4}\right)\right)$$

where ${\uptheta }_{1}$ is the parameter corresponding to the reference group (treatment arm one) and is equivalent to the intercept parameter ${\upbeta }_{0}$ in the GLM framework, ${X}_{m2},{X}_{m3},{X}_{m4}$ are dichotomous variables indicating membership to treatment arms two, three, and four, respectively, and **W** is a matrix with K (total number of clusters) columns of dichotomous variables indicating membership to the *m*th cluster.

Draws from the posterior distribution were obtained using the No-U-Turn-Sampler as is standard in Stan. For each model, 4 chains were used, with each chain consisting of 750 warmups and 750 draws, for a total of 3,000 warmups and 3,000 draws. The ‘adapt_delta’ (Δ) is the target average acceptance probability in the Stan sampling [31]. The closer the Δ to one, the smaller the steps the sampler takes, increasing computation time but also increasing the robustness of the sampler. A Δ of 0.99 was used, instead of the default 0.8 due to the small number of clusters per arm and participants per cluster. The parameters were non-centered to increase the likelihood of convergence; this approach is crucial for improving convergence in hierarchical models when the data are weak [34].

The ‘future’ package in R was used to run the multi-core simulations [35]. With the ‘future package’, a seed set within the ‘future’ function ensures that the random generation is forwarded one step in the random number generator state as to not duplicate the random generation.

### Number of repetitions

A total 2,500 simulations were performed to evaluate each trial property combination. In a worst-case scenario, with 50% power the Monte Carlo standard error would be 0.01 (1%) with 2,500 simulations (Eq. 2) [27].

2 $$\begin{aligned}\frac{\widehat{power} \ast (1-\widehat{power})}{MCS{E}^{2}}&={n}_{sim} \\ \frac{0.5\ast 0.5}{{0.01}^{2}}&=2500\end{aligned}$$

### Adaptions

This simulation study considered two adaptions in particular: arm dropping and early stopping for futility. Simulated trials that used adaptive designs included both the early stopping for futility and the arm dropping designs.

Arm dropping is the process of stopping recruitment to treatment arms if sufficient evidence of their lack of efficacy is gathered. By dropping poorly performing arms and assigning the clusters to the remaining arms, power to assess the effects of the remaining arms can be improved. Stopping for futility is the interim decision to stop the trial early and conclude there is sufficient evidence of a lack of efficacy of the treatment(s). Using this adaptive design can minimise trial cost and time.

A treatment arm was considered for dropping if its Bayesian posterior probability of being the arm with the highest effect size was $\text{<}0.05$ (Eq. 3). The control arm could not be dropped. If multiple treatment arms could be dropped, then the treatment arm with the lowest posterior probability was dropped. If the posterior probability of success was tied the arm with the lowest effect estimate was dropped.

3 $$\text{For j in 2, 3, 4: } P\left({\theta }_{j}=max\left\{{\theta }_{1},{\theta }_{2},{\theta }_{3},{\theta }_{4}\right\}\right|data)<0.05$$

A trial was stopped early for futility if every treatment arm had a posterior probability of being the best arm < 0.3 (Eq. 4). If both early stopping and arm dropping criteria were met, then the trial was stopped early. The adaptive interim decisions made by trials when using different cut-points for stopping early for futility and arm dropping are presented in Supplementary Files 1 and 2, respectively.

4 $$\begin{aligned}&P\left(\theta_2=max(\theta_j)\vert data\right)<0.3\:\&\\&P\left(\theta_3=max(\theta_j)\vert data\right)<0.3\:\&\\&P\left(\theta_4=max(\theta_j)\vert data\right)<0.3\end{aligned}$$

For trials using adaptive designs, interim analysis was performed when the number of recruited clusters reached a minimum 50% of the final cluster number. This meant, for the trials with 5 clusters per arm, an interim analysis was conducted at three clusters per arm recruited, and for the trials with 10 clusters per arm the interim analysis was conducted at 5 clusters per arm recruited.

### Performance measures

We assessed the power, type 1 error, and convergence. While type 1 error and power can be thought of as traditionally frequentist notions, these performance measures are often used and recommended [36] in trials using a Bayesian framework [37].

For each trial property combination and non-null scenario, the power was defined as the proportion of successful trials.

Let ${\theta }_{j}$ be treatment effect estimates for treatment arms $j=1, 2,\dots ,j$. We considered $j=4$ arms. For the non-null scenarios, the trial was considered a success if the proportion of posterior draws with ${\theta }_{4}$ (the treatment effect of the optimal arm) as the largest treatment effect was $\ge 0.95$ (see Eq. 5).

5 $$P\left({\theta }_{4}=max\left\{{\theta }_{j}\right\}\right|data)\ge 0.95$$

For each trial property combination, type 1 error was defined the proportion of trial successes in the null scenario. In the null scenario, the trial was deemed a (incorrect) success if the proportion of posterior draws with ${\theta }_{j}$ as the largest treatment effect was $\ge 0.95$ (see Eq. 6).

6 $$\begin{aligned}&P\left({\theta }_{2}=max\left\{{\theta }_{j}\right\}\right|data)\ge 0.95 \text{ or } \\&P\left({\theta }_{3}=max\left\{{\theta }_{j}\right\}\right|data)\ge 0.95 \text{ or } \\&P\left({\theta }_{4}=max\left\{{\theta }_{j}\right\}\right|data)\ge 0.95\end{aligned}$$

The power and type 1 error under these trial conditions was calculated and graphed over the design space and compared across the candidate designs. In addition, the proportion of simulated trials that stopped early for futility, and the proportion of trials that dropped a treatment arm and which arm was dropped were graphed over the trial properties.

The $\widehat{R}$ is the ratio of the standard deviation of the estimate total and the within chain standard deviation [38]. The $\widehat{R}$ used in Stan is the maximum of the rank normalised split-r-hat and rank normalised-folded-split-R-hat [31]. An $\widehat{R}$ > 1.05 means that the estimates’ chains don’t agree, which could indicate unreliable estimates. The ESS of the bulk and tail measures how efficient the sampling of the bulk and tails of the estimates’ distribution are, respectively. An ESS < 100 per chain is indicative that the sampling of that part of the distribution is not efficient, resulting in potential poor model fit. To assess convergence, the proportion of simulations with an $\widehat{R}$ > 1.05 or an effective sample size (ESS) bulk or tail < 400 in any treatment arm (non-reference) estimate was tabulated by the trial properties and adaptions.

### Pilot

A pilot of the non-adaptive trial was run. For each trial property combination three simulations were run. Prior predictive checks for each of these simulations were performed by drawing from the prior and comparing the sampled prior values to the true prior and the posterior values on a bar chart. The posterior checks were performed by making trace plots using the ‘rstan’ package [39]. The $\widehat{R}$ and ESS of the bulk and tail were measured.

## Results

### Convergence

Across all trial properties including in the interim analysis (with 3 and 5 clusters per arm), the proportion of trials with ESS bulk < 400, ESS tail < 400, or $\widehat{R}$ > 1.05 was < 0.02 (2%) indicating sound model convergence.

### Trial adaptive decisions

**Fig. 1:**
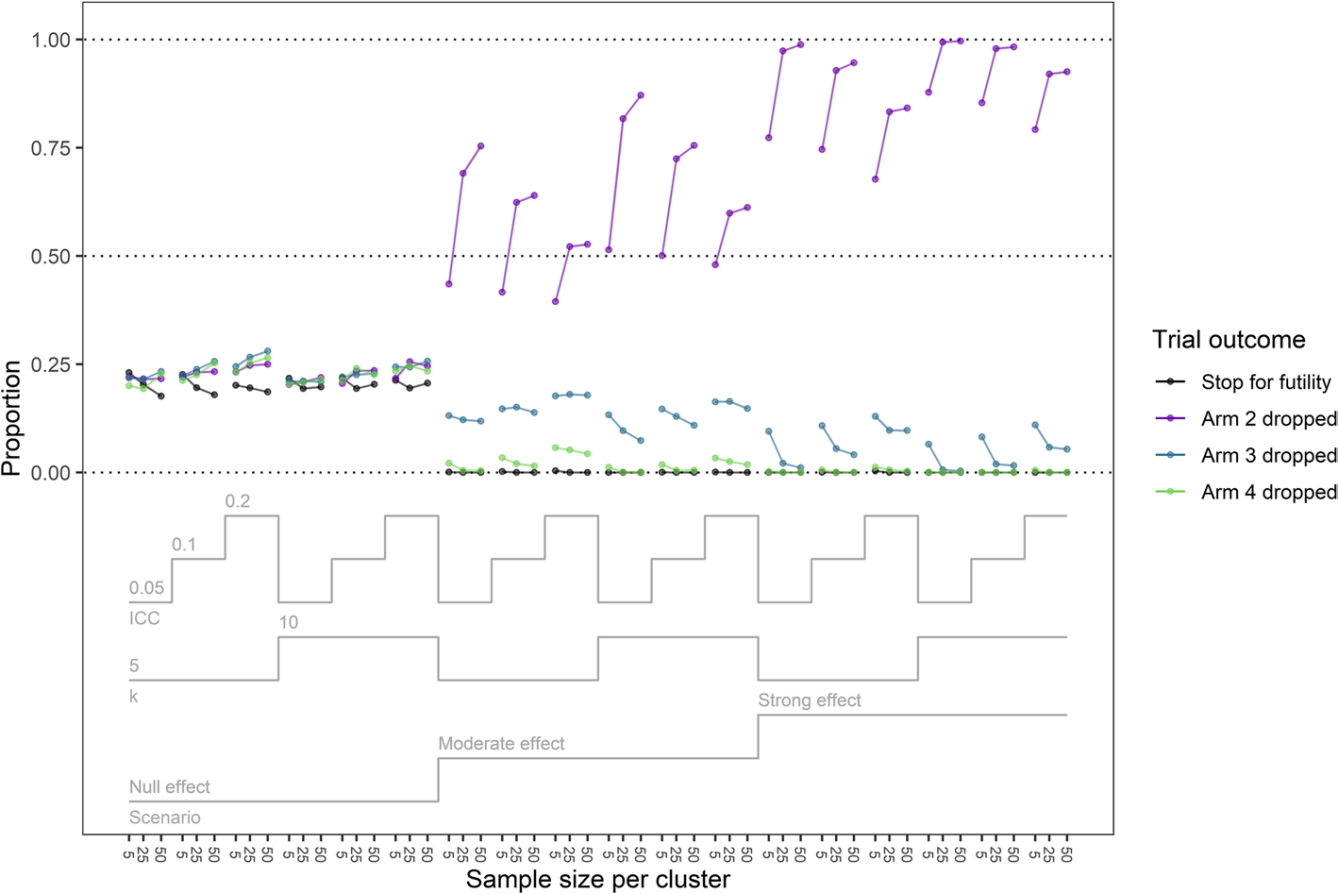
Proportion of trials that dropped an arm or stopped for futility by trial properties

Figure 1 details the adaptive interim decisions made across trial properties. Supplementary Files 1 and 2 contain the proportion of trials that dropped a treatment arm or stopped for futility using different cutpoints. In the non-null scenarios < 0.1% of trials stopped for futility. In the null scenario 17.6% to 23.1% of trials stopped for futility, and the proportion of trials that stopped for futility or dropped an arm was higher when the ICC = 0.2. When the ICC was high, the proportion of trials that dropped Arm 4 in the non-null scenarios was also higher (range = 0%—5.7%) than when the ICC was moderate (0%—3.4%) or low (range = 0%—2.1%). Arm 4 was also more likely to be dropped when the number of participants per cluster was lower, the number of clusters per arm was smaller, and when the effect size was smaller.

In the non-null scenarios, as the number of participants per cluster increased from 5 to 25, the proportion of trials that dropped Arm 2 (the least effective treatment arm) increased notably (range = 39.5%—87.8% vs 52.2%—99.4%). This increase was not as pronounced when the number of participants increased from 25 to 50, with the proportion of trials dropping Arm 2 only increasing to a range of 52.7%—99.6%. The proportion of trials that dropped Arm 2 also increased when the number of clusters per arm increased from 5 (range = 39.5%—98.8%) to 10 (range = 48.0%—99.6%) and in Scenario 1 compared to Scenario 2 (range = 39.5%—87.1%) to large (range = 67.7%—99.6%).

### Power and type 1 error

**Fig. 2:**
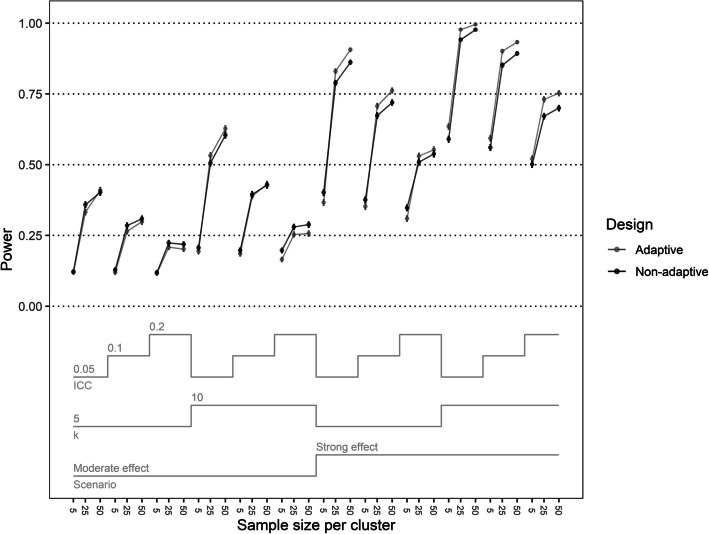
Power of the adaptive and non-adaptive designs over trial properties. Error bars represent the Monte Carlo standard error

**Fig. 3:**
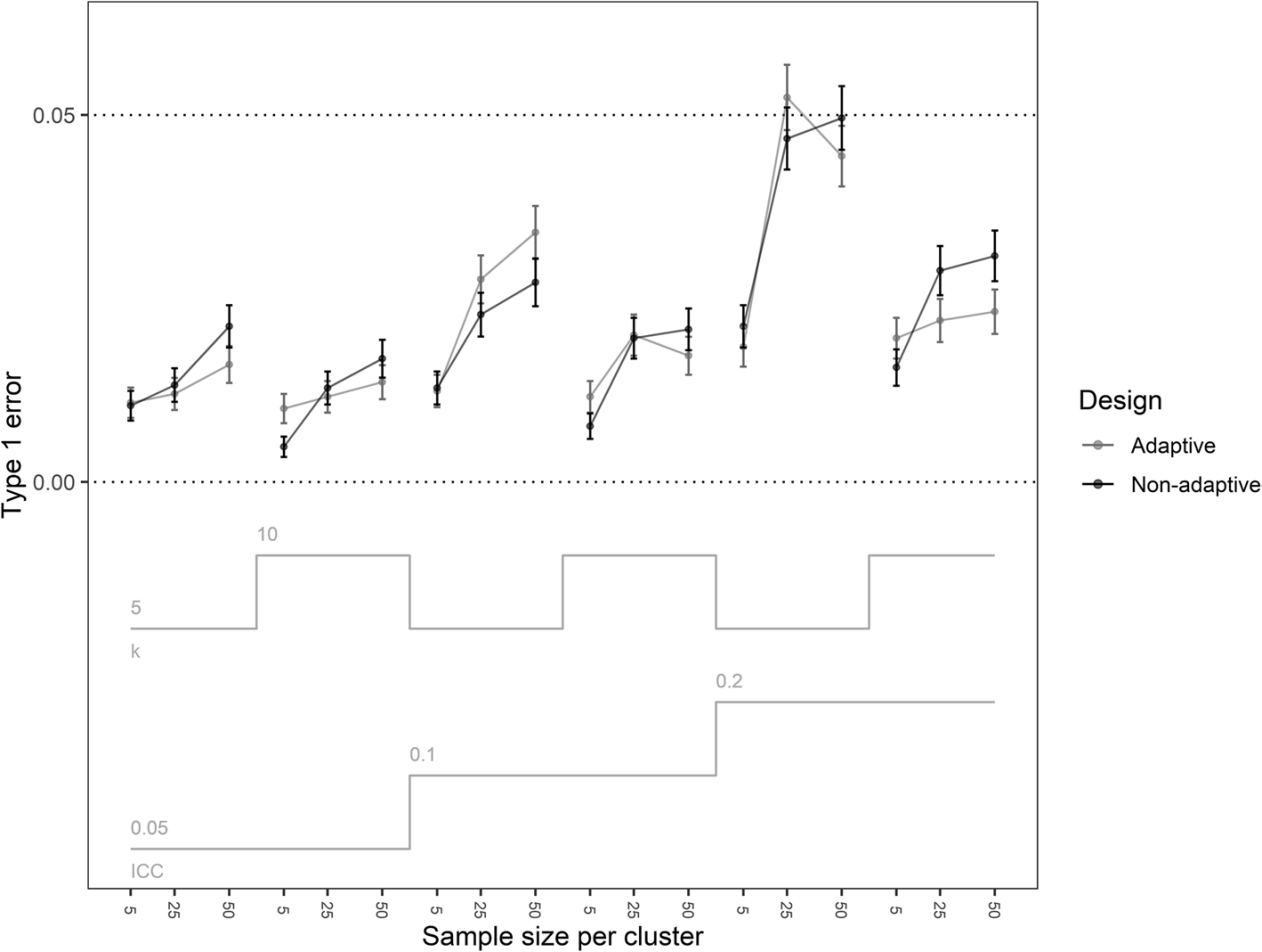
Type 1 error for the adaptive and non-adaptive designs over trial properties. Error bars represent the Monte Carlo standard error

Figures 2 and 3 present the power and type 1 error of the simulated trials, respectively. In Scenario 1, the adaptive design trials achieved higher power than the non-adaptive design trials except for when the number of participants per cluster = 5 and the number of clusters per arm = 5 (difference range = 0.015–0.059) (Fig. 2). In Scenario 2, trials using an adaptive design had higher power than non-adaptive design trials when the ICC = 0.05 and the total sample size per arm > = 250 (difference range = 0.007–0.026). Otherwise, the power of the adaptive design trials was nearly equivalent or lower to the non-adaptive design trials (difference range = −0.032 – −0.002).

The type 1 error ranged from 0.01 to 0.05, with higher type 1 errors in trials with ICC = 0.2 (Fig. 3). Notably, the combination of participants per cluster > 5, number of clusters per arm = 5, and ICC = 0.2 resulted in the largest type 1 errors for both the adaptive and non-adaptive trials (range = 0.044–0.052). Adaptive design trials had a lower type 1 error when the total number of participants per arm was > = 250, or when the total number of participants per arm = 125 and the ICC was 0.05 (difference range = −0.01 – −0.001).

## Discussion

We simulated the convergence, power, and type 1 error of cluster randomised control trials across trial properties, with and without adaptive designs.

We demonstrated that the power and type 1 error of adaptive designs using one interim analysis is comparable and sometimes better performing to non-adaptive designs. The adaptive design trials had marginally increased power compared to the non-adaptive trials across most design spaces and the type 1 error was comparable or slightly lower in all design spaces. The comparable type 1 error is not unexpected, as the stopping for futility adaption is known to lower the risk of making a type 1 error, but this change in risk is minimal with 1 interim [40, 41]. The slightly increased power is also not unexpected, previous studies have demonstrated that adaptive randomisation (including arm dropping) has potential to increase power to remaining arms [41, 42]. Removing the worst performing arm and randomising the remaining clusters to the better performing arms means that the remaining arms have a smaller variance and thus are more likely to make a correct conclusion about the optimal arm instead of being inconclusive.

Adaptive designs do not increase the power equally across all trial properties. If ICC is expected to be high in a trial with a low sample size, then adaptive designs may not be ideal if the purpose of these designs are to increase power. The ICC had a notable effect on the power improvement of adaptive designs with a small number of participants per cluster and number of clusters per arm, with a high ICC resulting in worse power for adaptive designs than non-adaptive in some cases. This higher ICC is seen in implementation trials, especially when one participants’ outcome can be highly affected by another participants’ outcome (e.g. one participant encouraging the other to join in activity) [4, 43]. Higher ICCs have been observed, such as in the trial by Hall et al*.* (2022), where the ICC reached 0.384 for student level outcomes [43]. At present adaptive trials maybe most beneficial when the primary outcome is expected to have a lower ICC or a larger sample size, for trials with primary outcomes with high levels of clustering adaptive designs are no more appropriate than non-adaptive designs.

When the ICC was large and number of clusters per arm was low, the type 1 error slightly rose with an increasing number of participants per cluster. This type 1 error was observed with a static cut-point of success at 0.95. There are some methods to alleviate a high type 1 error. Typically, to reduce type 1 error to an acceptable level the cut-point of success should be increased, but this also reduces the power. Trying to determine the ideal cut-point for declaring success, dropping an arm, or stopping for futility is a balancing act between achieving adequate power and keeping the type 1 error at or below 0.05. To determine the ideal cut-point to manage type 1 error, a simulation of the proposed trial can be run [44]. Modelling the trial results under a Bayesian framework can also reduce the type 1 error rates [41, 45]. In particular, hierarchical Bayesian models are noted to have lower type 1 error rates likely due to partial pooling [46, 47]. It is not known whether the use of a hierarchical model in our simulations lowered the type 1 error as we did not compare to a non-hierarchical model, but we did observe that the high ICC and small sample sizes attenuated the benefits from our hierarchical model on the type 1 error and power. This likely occurred as the less uncertainty within a cluster (and thus the higher the ICC), the less shrinkage that occurs [48, 49]. This means that less information is being borrowed from other clusters within that treatment arm, reducing the pooling that occurs.

The convergence of the models and adaptive interim decisions were promising, which leads to the conclusion that adaptive designs were robust for use in trials seeking to optimise implementation strategies. The adaptive interim decisions rarely were unfavourable, with trials in the non-null scenario declaring futility in less than 0.01% of trials or dropping the optimal arm in less than 6% of trials. There were instances where the unfavourable decisions were made more frequently, namely when the ICC was 0.2. In situations where researchers predict the ICC may be high for outcomes being used for adaptive decisions, adaptive designs may not be feasible due to the higher risk of unfavourable/incorrect decisions.

The model convergence was sound, indicating that even at small sample and cluster sizes seen in interim analyses assessing optimisation is statistically feasible. Across all trial properties for the adaptive and non-adaptive designs the ESS bulk, ESS tail, and $\widehat{R}$ values indicated that the sampling in the bulk and tail of the distribution was efficient and that there was agreement between chains. To improve model convergence, increasing the $\Delta$ and maximum tree depth, simplifying the model (i.e., by removing covariates and/or random effects, if possible) and, uncentering the treatment effects are all recommended [31, 50]. If the ESS is < 400 (100 per chain), sampling of the posterior distribution is less efficient. Using more warmups and draws may alleviate this, as well as using more informative priors for any/all effects. Until then, the $\widehat{R}$ can be unreliable [38]. The choice of prior for variance component has been discussed in detail elsewhere, but in summary for a low number of clusters the half normal distribution performs well [51] and choose a choose a variance smaller than the effect SD improves convergence [52]. Cluster level covariates are also an important consideration. If cluster level covariates aren’t balanced (likely for a small number of clusters per arm) or randomisation is stratified by these covariates, they need to be accounted for in the model. However, with a small numbers of clusters per arm, the model may not be able to effectively control these differences, especially if there’s many covariates to adjust.

An important note for the practical feasibility of adaptive designs for trials seeking to optimise is that they can introduce a variable cost of the overall trial. For example, if a lower cost arm is dropped in the trial, clusters will subsequently be randomised to higher cost arms thus making total cost of the trial increase past the initial expectations. It is recommended to provide a worst-case scenario in terms of cost or include a plausible range of values.

### Limitations

A limitation of the study was that the model simulated was not complicated, with only variables for cluster and treatment arm. We aimed to initially explore the statistical feasibility of adaptive designs with one of the models that showed the most promise for statistical feasibility. If this initial model was unfeasible, it was unlikely that more complicated models would be statistically feasible. How the models would converge with interaction effects, effects for time and participant are not known, but increased model complexity could likely cause issues with the smaller arm and cluster sizes as it will be harder to explore the full parameter space. This may be an issue for the frequently recommended (in the optimisation field) factorial trial design, where the model can include multiple interaction effects. Future research should explore if adaptive designs remain statistically feasible with more complicated models and consider the minimum number of clusters per arm required for adaptive designs to be viable.

Obtaining information on how the different adaptive designs could affect the trial outcomes was limited with only one interim analysis. There would be more chances for the adaptions to take place if there were more interims. In addition, only a relatively small set of values for design parameters were explored. This limits the generalisability of the results. Future research could study the effects of adaptive designs when run over a larger trial with more interim analyses, and with addition design parameters. This study only considered the conservative approach of dropping one arm at a time, but in practice the approach could be extended to dropping more than one arm at interim if practical.

In this simulation we made all the clusters receive the treatment effects equally, but this may not reflect real world features. With a low number of clusters per arm there’s likely to be imbalance between cluster level confounders. If this occurs, then one may need include those confounders in the model, potentially introducing sampling difficulties.

Simulating the binomial response in a cluster design using a random intercept to model the cluster resulted in an upwards bias. This biased increased for a higher ICC. This bias occurred as the random effects model was modelling the subject-specific effects, while we were trying to simulate the population level effects [53]. Simulating the data with a generalised estimating equation could remove this bias as it models the population level effects, but it does not perform well with a small number of clusters per arm as the type I error rate is typically inflated beyond the nominal level [54]. Since all arms had the same level of bias, it is expected the effect on power and type 1 error was minimal.

## Conclusion

Adaptive designs are statistically feasible for use and can provide small power increases to multi-arm trials aiming to optimise implementation strategies without increasing the type 1 error. However, when the ICC of the outcome of interest in a trial is expected to be high (for example, ICC = 0.2), adaptive designs have reduced statistical feasibility due to an increased risk of incorrect adaptive interim decisions being made, but can still provide power gains without increasing the type 1 error.

## Supplementary Information

Supplementary Material 1.

Supplementary Material 2.

## Data Availability

Code for the simulations is located at https://github.com/ErnKNolan/PhDSims/. Simulated datasets are located at https://osf.io/msau7/. Results were generated using R version 4.3.1, cmdstanr version 0.6.1, and Stan version 2.26.1.

## Abbreviations

ADEMP: Aims, data generating mechanisms, estimands, methods, performance measures
BASIS: Bayesian simulation study
ESS: Effective sample size
ICC: Intra-class correlation
RCT: Randomised control trial
