# Supplementary material for: Properties of adaptive, cluster-randomised controlled trials with few clusters: a simulation study
Source: Implement Sci. 2025 Jul 1;20:31. doi: 10.1186/s13012-025-01443-6 (PMC12211755; doi:10.1186/s13012-025-01443-6)
Supplement: Supplementary file 1 — Supplementary Material 1. [file 13012_2025_1443_MOESM1_ESM.docx]

Supplementary file 1: Proportion of trials that stopped for futility by trial properties and futility cut-point.

| **Trial properties** | | | | **Cut-point for futility** | | | | |
| --- | --- | --- | --- | --- | --- | --- | --- | --- |
| **Scenario** | **ICC** | **n per k** | **k** | **0.15** | **0.20** | **0.25** | **0.35** | **0.45** |
| Strong effect | 0.05 | 5 | 5 | 0 | 0 | 0 | 0.002 | 0.018 |
| Strong effect | 0.05 | 5 | 10 | 0 | 0 | 0 | 0.001 | 0.005 |
| Moderate effect | 0.05 | 5 | 5 | 0 | 0 | 0 | 0.015 | 0.073 |
| Moderate effect | 0.05 | 5 | 10 | 0 | 0 | 0 | 0.004 | 0.045 |
| No effect | 0.05 | 5 | 5 | 0.066 | 0.098 | 0.154 | 0.294 | 0.460 |
| No effect | 0.05 | 5 | 10 | 0.055 | 0.099 | 0.145 | 0.295 | 0.470 |
| Strong effect | 0.2 | 5 | 5 | 0.001 | 0.001 | 0.002 | 0.008 | 0.035 |
| Strong effect | 0.2 | 5 | 10 | 0 | 0 | 0 | 0.001 | 0.009 |
| Moderate effect | 0.2 | 5 | 5 | 0 | 0.000 | 0.002 | 0.016 | 0.075 |
| Moderate effect | 0.2 | 5 | 10 | 0 | 0 | 0.001 | 0.008 | 0.051 |
| No effect | 0.2 | 5 | 5 | 0.075 | 0.108 | 0.148 | 0.260 | 0.400 |
| No effect | 0.2 | 5 | 10 | 0.070 | 0.117 | 0.157 | 0.267 | 0.407 |
| Strong effect | 0.05 | 25 | 5 | 0 | 0 | 0 | 0 | 0 |
| Strong effect | 0.05 | 25 | 10 | 0 | 0 | 0 | 0 | 0 |
| Moderate effect | 0.05 | 25 | 5 | 0 | 0 | 0 | 0.001 | 0.020 |
| Moderate effect | 0.05 | 25 | 10 | 0 | 0 | 0 | 0 | 0.006 |
| No effect | 0.05 | 25 | 5 | 0.067 | 0.108 | 0.154 | 0.264 | 0.411 |
| No effect | 0.05 | 25 | 10 | 0.062 | 0.095 | 0.142 | 0.256 | 0.409 |
| Strong effect | 0.2 | 25 | 5 | 0 | 0 | 0 | 0 | 0.006 |
| Strong effect | 0.2 | 25 | 10 | 0 | 0 | 0 | 0 | 0.002 |
| Moderate effect | 0.2 | 25 | 5 | 0 | 0 | 0 | 0.001 | 0.034 |
| Moderate effect | 0.2 | 25 | 10 | 0 | 0 | 0 | 0 | 0.024 |
| No effect | 0.2 | 25 | 5 | 0.097 | 0.131 | 0.161 | 0.235 | 0.344 |
| No effect | 0.2 | 25 | 10 | 0.085 | 0.123 | 0.158 | 0.233 | 0.357 |
| Strong effect | 0.05 | 50 | 5 | 0 | 0 | 0 | 0 | 0 |
| Strong effect | 0.05 | 50 | 10 | 0 | 0 | 0 | 0 | 0 |
| Moderate effect | 0.05 | 50 | 5 | 0 | 0 | 0 | 0 | 0.012 |
| Moderate effect | 0.05 | 50 | 10 | 0 | 0 | 0 | 0 | 0.002 |
| No effect | 0.05 | 50 | 5 | 0.062 | 0.093 | 0.130 | 0.229 | 0.382 |
| No effect | 0.05 | 50 | 10 | 0.070 | 0.107 | 0.148 | 0.251 | 0.406 |
| Strong effect | 0.2 | 50 | 5 | 0 | 0 | 0 | 0 | 0.004 |
| Strong effect | 0.2 | 50 | 10 | 0 | 0 | 0 | 0 | 0 |
| Moderate effect | 0.2 | 50 | 5 | 0 | 0 | 0 | 0.001 | 0.030 |
| Moderate effect | 0.2 | 50 | 10 | 0 | 0 | 0 | 0 | 0.022 |
| No effect | 0.2 | 50 | 5 | 0.094 | 0.122 | 0.153 | 0.220 | 0.314 |
| No effect | 0.2 | 50 | 10 | 0.088 | 0.128 | 0.168 | 0.252 | 0.370 |

ICC = intra-class correlation, n per k = number of participants per cluster, k = number of clusters per arm
