# Supplementary material for: Properties of adaptive, cluster-randomised controlled trials with few clusters: a simulation study
Source: Implement Sci. 2025 Jul 1;20:31. doi: 10.1186/s13012-025-01443-6 (PMC12211755; doi:10.1186/s13012-025-01443-6)
Supplement: Supplementary file 2 — Supplementary Material 2. [file 13012_2025_1443_MOESM2_ESM.docx]

Supplementary file 2: Proportion of trials that dropped each treatment arm by trial properties and arm dropping cut-point.

| **Trial properties** | | | | **Cut-point = 0.10** | | | **Cut-point = 0.15** | | |
| --- | --- | --- | --- | --- | --- | --- | --- | --- | --- |
| **Scenario** | **ICC** | **n per k** | **k** | **Arm 2** | **Arm3** | **Arm 4** | **Arm 2** | **Arm 3** | **Arm 4** |
| Strong effect | 0.05 | 5 | 5 | 0.829 | 0.114 | 0.004 | 0.846 | 0.124 | 0.006 |
| Strong effect | 0.05 | 5 | 10 | 0.904 | 0.075 | 0.001 | 0.912 | 0.078 | 0.001 |
| Moderate effect | 0.05 | 5 | 5 | 0.539 | 0.188 | 0.041 | 0.596 | 0.220 | 0.054 |
| Moderate effect | 0.05 | 5 | 10 | 0.616 | 0.178 | 0.022 | 0.662 | 0.208 | 0.035 |
| No effect | 0.05 | 5 | 5 | 0.283 | 0.276 | 0.261 | 0.315 | 0.302 | 0.288 |
| No effect | 0.05 | 5 | 10 | 0.268 | 0.278 | 0.265 | 0.304 | 0.312 | 0.292 |
| Strong effect | 0.2 | 5 | 5 | 0.737 | 0.166 | 0.017 | 0.756 | 0.178 | 0.022 |
| Strong effect | 0.2 | 5 | 10 | 0.828 | 0.122 | 0.008 | 0.840 | 0.130 | 0.008 |
| Moderate effect | 0.2 | 5 | 5 | 0.478 | 0.229 | 0.088 | 0.514 | 0.262 | 0.109 |
| Moderate effect | 0.2 | 5 | 10 | 0.558 | 0.213 | 0.055 | 0.598 | 0.236 | 0.072 |
| No effect | 0.2 | 5 | 5 | 0.284 | 0.297 | 0.281 | 0.308 | 0.322 | 0.305 |
| No effect | 0.2 | 5 | 10 | 0.277 | 0.292 | 0.289 | 0.303 | 0.318 | 0.314 |
| Strong effect | 0.05 | 25 | 5 | 0.977 | 0.022 | 0 | 0.978 | 0.022 | 0 |
| Strong effect | 0.05 | 25 | 10 | 0.994 | 0.006 | 0 | 0.994 | 0.006 | 0 |
| Moderate effect | 0.05 | 25 | 5 | 0.752 | 0.144 | 0.011 | 0.782 | 0.158 | 0.016 |
| Moderate effect | 0.05 | 25 | 10 | 0.852 | 0.107 | 0.001 | 0.863 | 0.114 | 0.001 |
| No effect | 0.05 | 25 | 5 | 0.279 | 0.289 | 0.259 | 0.310 | 0.324 | 0.291 |
| No effect | 0.05 | 25 | 10 | 0.269 | 0.271 | 0.276 | 0.305 | 0.305 | 0.303 |
| Strong effect | 0.2 | 25 | 5 | 0.859 | 0.107 | 0.007 | 0.867 | 0.110 | 0.010 |
| Strong effect | 0.2 | 25 | 10 | 0.928 | 0.062 | 0 | 0.932 | 0.064 | 0 |
| Moderate effect | 0.2 | 25 | 5 | 0.585 | 0.211 | 0.068 | 0.612 | 0.234 | 0.079 |
| Moderate effect | 0.2 | 25 | 10 | 0.658 | 0.193 | 0.037 | 0.684 | 0.206 | 0.045 |
| No effect | 0.2 | 25 | 5 | 0.287 | 0.311 | 0.296 | 0.304 | 0.330 | 0.316 |
| No effect | 0.2 | 25 | 10 | 0.302 | 0.288 | 0.294 | 0.326 | 0.310 | 0.315 |
| Strong effect | 0.05 | 50 | 5 | 0.989 | 0.011 | 0 | 0.989 | 0.011 | 0 |
| Strong effect | 0.05 | 50 | 10 | 0.996 | 0.004 | 0 | 0.996 | 0.004 | 0 |
| Moderate effect | 0.05 | 50 | 5 | 0.798 | 0.135 | 0.006 | 0.814 | 0.146 | 0.010 |
| Moderate effect | 0.05 | 50 | 10 | 0.895 | 0.082 | 0.001 | 0.904 | 0.085 | 0.002 |
| No effect | 0.05 | 50 | 5 | 0.262 | 0.290 | 0.295 | 0.289 | 0.316 | 0.324 |
| No effect | 0.05 | 50 | 10 | 0.281 | 0.280 | 0.273 | 0.308 | 0.308 | 0.303 |
| Strong effect | 0.2 | 50 | 5 | 0.860 | 0.108 | 0.006 | 0.869 | 0.111 | 0.006 |
| Strong effect | 0.2 | 50 | 10 | 0.935 | 0.057 | 0 | 0.940 | 0.058 | 0 |
| Moderate effect | 0.2 | 50 | 5 | 0.584 | 0.219 | 0.063 | 0.610 | 0.241 | 0.073 |
| Moderate effect | 0.2 | 50 | 10 | 0.679 | 0.179 | 0.030 | 0.710 | 0.195 | 0.038 |
| No effect | 0.2 | 50 | 5 | 0.284 | 0.318 | 0.304 | 0.303 | 0.335 | 0.322 |
| No effect | 0.2 | 50 | 10 | 0.290 | 0.301 | 0.274 | 0.313 | 0.329 | 0.300 |

ICC = intra-class correlation, n per k = number of participants per cluster, k = number of clusters per arm
